# Online Video Instruction on Hand Expression of Colostrum in Pregnancy is an Effective Educational Tool

**DOI:** 10.3390/nu11040883

**Published:** 2019-04-19

**Authors:** Therese A. O’Sullivan, Joy Cooke, Chris McCafferty, Roslyn Giglia

**Affiliations:** 1 School of Medical and Health Sciences, Edith Cowan University, 270 Joondalup Drive, Joondalup, WA 6027, Australia; 2 Maternity Unit, Glengarry Private Hospital, Duncraig, WA 6023, Australia; joy.cooke@westnet.com.au; 3 School of Nursing and Midwifery, Edith Cowan University, Joondalup, WA 6027, Australia; c.mccafferty@ecu.edu.au; 4 Telethon Kids Institute, University of Western Australia, Subiaco, WA 6008, Australia; roslyn.giglia@telethonkids.org.au

**Keywords:** antenatal, expressing, pregnancy, video instruction, colostrum, breastfeeding, online

## Abstract

The use of antenatal colostrum expression in the weeks prior to birth may help improve long-term breastfeeding, but few large-scale studies exist. Typically, antenatal colostrum expression instruction relies on face-to-face education, making large interventions costly. We aimed to determine whether an expert online instructional video can improve knowledge and confidence around antenatal colostrum expressing. Pregnant women were asked to complete a questionnaire pre- and post-watching the instructional video online. Ninety five pregnant women completed both pre- and post-questionnaires. Total antenatal colostrum expression knowledge scores improved after watching the video, from a mean of 3.05 ± 1.70 correct out of a maximum of 7, to 6.32 ± 0.76 (*p* < 0.001). Self-reported confidence around hand expressing in pregnancy also improved from an average ranking of not confident (2.56 ± 1.17, out of a possible 5) to confident (4.32 ± 0.80, *p* < 0.001). Almost all women (98%) reported that they would recommend the video to a friend or family member if antenatal colostrum expression was suggested by their healthcare provider. Findings suggest that the use of an online expert video is an acceptable and effective way to educate pregnant women in antenatal colostrum expression.

## 1. Introduction

During pregnancy, breasts begin to produce the first milk, colostrum. Historically, antenatal colostrum expression (ACE) was performed as a means of preparing the breasts for breastfeeding after birth. More recently, ACE is performed to collect a supply of colostrum. Colostrum can be collected using a syringe, and safely stored in a freezer. The colostrum can then be defrosted and given to the baby. The stored colostrum can be used to treat hypoglycaemia in the infant after birth, or if breastfeeding issues occur. The collection and storage of colostrum can ensure that an infant receives its own mother’s milk as the first feed after birth, instead of reliance on formula during this time. In women intending to exclusively breastfeed, formula supplementation while in hospital has been associated with a two- to three-fold risk of early breastfeeding cessation [1]. Some have proposed that ACE can also promote the onset of Lactogenesis II, the “coming in” of breastmilk, however there is no evidence to support this effect, and the efficacy of the ACE practice is yet to be established in this regard [2].

In addition, there has been some concern that antenatal nipple stimulation while teaching hand expression will act as a precursor for the release of oxytocin, and may induce premature labour [3]. In a Cochrane review of the literature [4], breast stimulation was shown to reduce the number of women in labour after 72 h (i.e., had given birth), along with reduced postpartum haemorrhage rates. However, the authors went on to recommend not promoting this practice in high-risk women and noted that more information on maternal satisfaction was required. More recently, evidence suggests a role for nipple stimulation at term as a non-pharmacological method of labour induction [5,6]. In 2017, results of the Diabetes and Antenatal Milk Expressing (DAME) trial of 635 pregnant women with low-risk diabetes demonstrated that antenatal expressing did not result in a lower gestational age or increased admissions to the Neonatal Intensive Care Unit [7]. The authors concluded that there is no harm in advising women with diabetes in pregnancy at low risk of complications to express colostrum for the last few weeks of gestation. Other results from the DAME trial show a non-significant trend toward improvements in breastfeeding outcomes for women in the antenatal expressing intervention group, although the study was not powered for breastfeeding outcomes, and indicators for collecting infant feeding methods differed from standardised national indicators [8].

The question remains as to the effectiveness of ACE in promoting positive breastfeeding outcomes, and the safety of its practice as part of regular antenatal education at 37 weeks gestation in healthy, low-risk pregnant women. In our own medical record audit of a secondary, general hospital in Greater Perth, Western Australia, we compared the inclusion of ACE in antenatal education in relation to birthing outcomes in a hospital which had previously not included ACE in this education, using a retrospective control group. Results showed that inclusion of ACE in antenatal care was not associated with an increased risk of premature birth or admission to special care nursery [9].

Very few randomised controlled trials have been published in the area of ACE, with none in healthy women, and most using a small sample size (<100). Due to a lack of research investigating the safety and efficacy of antenatal colostrum expression in the general population, ACE is therefore not uniformly encouraged across Australian maternity health services. One major barrier to large scale trials in Australia is the cost of high-quality ACE instruction. A one-on-one session with a midwife (or lactation consultant) provides optimal ACE instruction, however it is not routinely taught as part of antenatal education. In our experience, detailed instruction and assistance with hand expressing is most often provided postnatally, commonly when women are experiencing breastfeeding difficulties (e.g., to relieve engorgement), are sleep deprived, and are potentially anxious about their ability to feed their babies.

**Figure A1:**
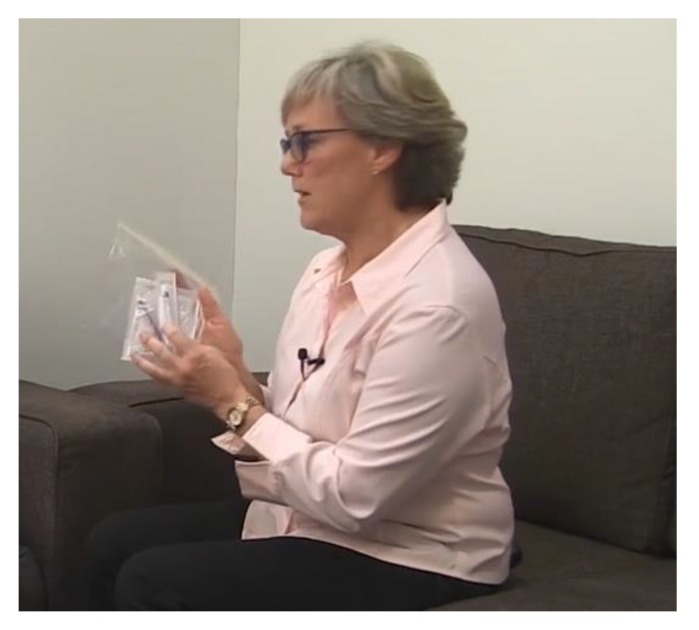
The lactation consultant discusses syringes and storage.

**Figure A2:**
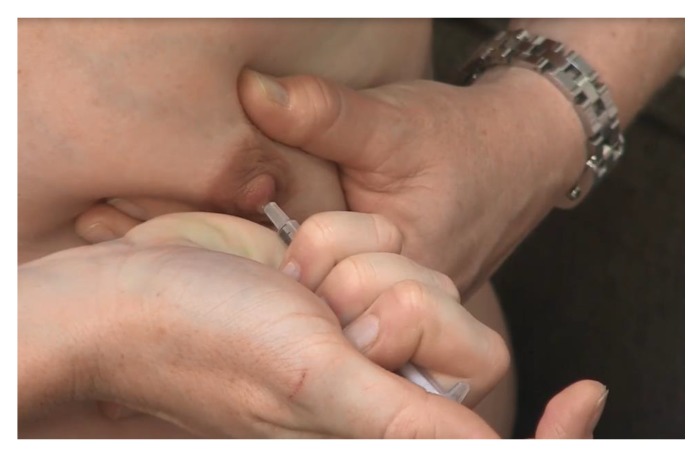
Close up of the colostrum collection during one of the demonstrations.

**Figure A3:**
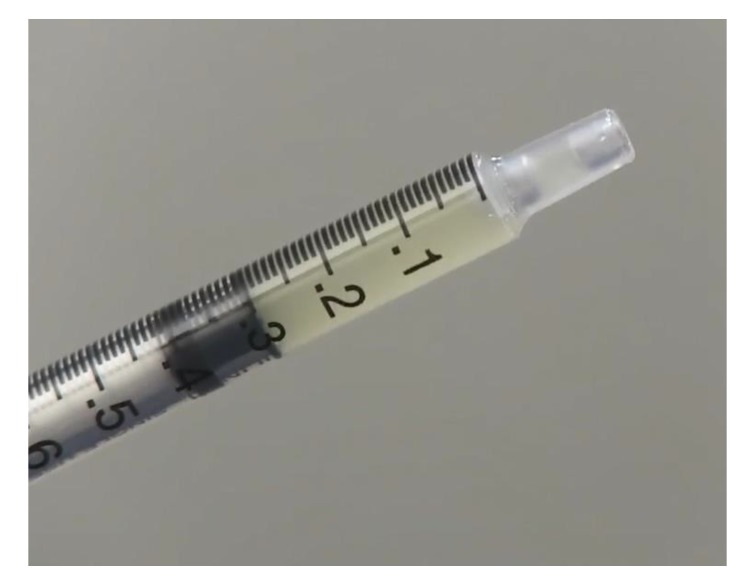
Close up of collected colostrum.

Online videos can be an effective and cheap tool for instruction, in areas where the internet is easily accessible. The expert expressing instructional videos that are currently available demonstrate breastmilk expressing in women who have already given birth. Given that expressing in postnatal women is conceptually different, this type of video is not well suited to an antenatal setting. Colostrum has a different look and consistency to mature milk, and the presence of the baby often stimulates milk “let down”. Feedback from West Australian midwives and lactation consultants, along with our local consumer reference group, indicated that a video of a lactation consultant-led demonstration of ACE using pregnant women would be very valuable. We have therefore developed an online instructional video on ACE specifically for pregnant women. To our knowledge, no such expert-informed video exists nationally or internationally that provides specific enough detail to suit requirements for use in a large-scale trial or in our health setting. The 20 min video featured a real-life situation, with two pregnant women in a session with a lactation consultant. The lactation consultant explained how to use ACE and how to collect and store the colostrum, and guided both women through the process in a practical demonstration (Appendix A, Figure A1, Figure A2 and Figure A3). The women had differing prior breastfeeding experience (one was primiparous, the other had previously breastfed multiple children), and differing breast sizes and nipples. The women also asked questions as they went through the process.

The aim of this study was to investigate whether the instruction on antenatal expression of colostrum via this instructional online video is an effective way to increase knowledge around ACE and confidence in using it, in pregnant women. If so, the use of an instructional video for ACE will provide an economical and feasible education option for future research.

## 2. Materials and Methods

### 2.1. Study Setting

This was a pre- and post-test study, using online questionnaires and video. Data collection for the study was completed online between August 2017 and April 2018.

### 2.2. Study Sample

Pregnant women were recruited through social media via local university and research institutions, and infant and mother organizations, along with personal contacts. The only inclusion criteria were being currently pregnant (any stage) and having access to the internet. Potential participants were provided with an online information letter and were requested to provide online consent prior to the start of the questionnaires. Ethics approval was granted from the Edith Cowan University Human Research Ethics Committee (17501).

### 2.3. Measurement

Two linked online questionnaires were developed using Qualtrics survey software (Provo, UT, USA). The first was a pre-video questionnaire which contained the ACE video at the end, followed by a second questionnaire which participants completed after viewing the video (post-video questionnaire). The pre-video questionnaire asked about age, gestation of current pregnancy, previous children and previous breastfeeding experience. The post-video questionnaire asked for feedback on the video, including aspects that were liked and disliked (free text response), the length of the video (too long, too short or appropriate), use of real pregnant women in the video (free text), and satisfaction on the depth of information covered, clarity of voices, image quality and overall satisfaction (assessed on a continuous 1–5 scale) from very unsatisfied to very satisfied. Respondents were also asked whether they would recommend the video to pregnant family and friends if ACE was suggested by their healthcare provider, and whether they had any suggestions for improvement of the video. Knowledge and confidence were assessed in both the pre- and post-questionnaires. Knowledge was assessed over seven areas and also as a total knowledge score (one point for each correct answer). To assess confidence about hand expressing in pregnancy, respondents were asked to move a sliding scale which ranged from 1–5, representing very unconfident to very confident.

### 2.4. Data Analysis

Descriptive statistics were used to report on subject characteristics, with means and standard deviations used for normally distributed results, and medians and interquartile ranges used to report results that were not normally distributed. The related samples McNemar statistical test was used to compare the number of correct answers in knowledge questions in the pre-video and post-video questionnaires, and the paired samples *t*-test was used to compare total knowledge scores and confidence around using ACE. Significance was set at *p* < 0.05.

For free text responses, information was grouped into themes and quotes that were identified as appropriate.

## 3. Results

A total of 171 pregnant women completed at least one aspect of the survey. Of these, 112 completed the full pre-questionnaire, with 95 going on to also complete the post-questionnaire after watching the video. Subject characteristics for the women who completed both questionnaires are shown in Table 1.

**Figure 1:**
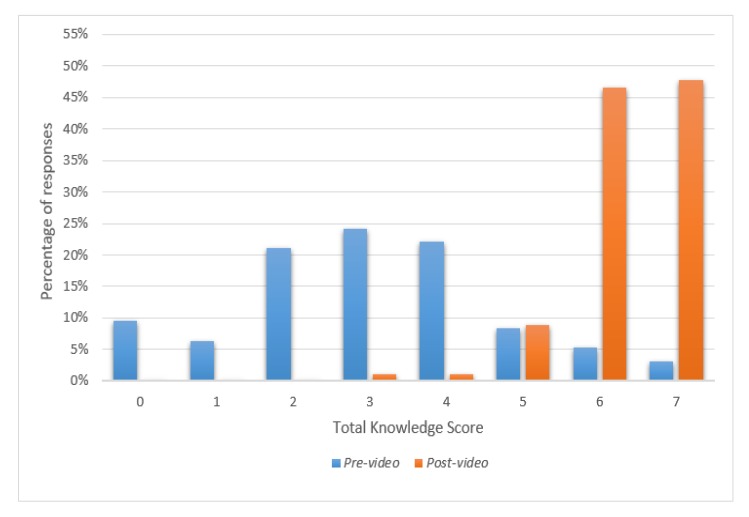
Chart of antenatal expressing of colostrum total knowledge scores scale pre- and post-video (minimum possible score = 0; maximum possible score = 7, *n* = 95).

**Figure 2:**
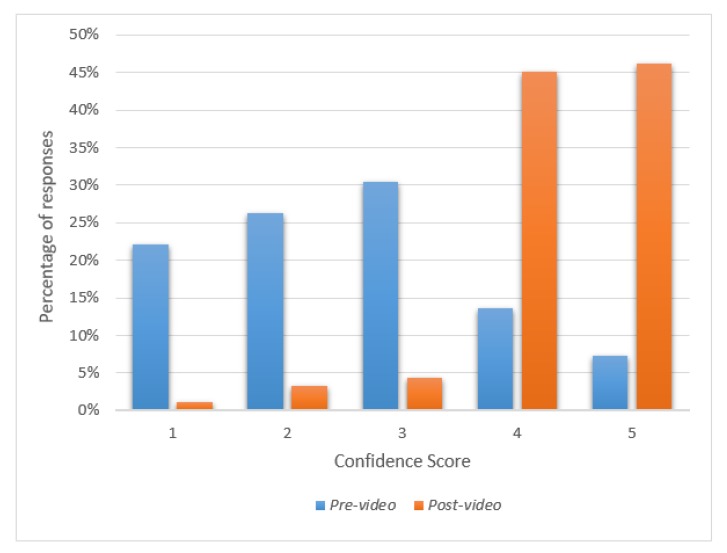
Chart of confidence scale pre- and post-video: How confident do you feel at the moment about hand expressing in pregnancy? (Minimum possible score = 1, representing very unconfident; maximum possible score = 5, representing very confident, *n* = 93).

Knowledge of ACE improved significantly for all areas after watching the video (Table 2), as did the total knowledge score (for all *p* < 0.001, Figure 1). Confidence in hand expressing during pregnancy also significantly increased from a mean score of 2.56 ± 1.17 to 4.32 ± 0.80 (*n* = 93, *p* < 0.001) (Figure 2). IOR: Inter quartile range.

When asked what they liked most about the video, 90 out of 95 respondents chose to write a full text response, which was subsequently grouped into one of five categories. The most common response was that the video was informative/interesting/detailed (32.6%). The inclusion of a practical demonstration (22.1%) in a real situation, and the use of real pregnant women (20.0%) were the next most frequently cited responses. Respondents also commented that the video was easy to understand and used clear explanations (15.8%). Others liked the video being “unrushed”, “comfortable” or “relaxed” (3.2%). One respondent answered that they liked “everything” (1.1%). Specific quotes relating to the responses are shown in Table 3.

When asked what they didn’t like about the video, the most common response was that it was too long (8.4%). This was followed by requests for more information (7.4%), including requests for information on why colostrum would be used, a visual example of when colostrum is not able to be collected using breast massage, when to stop collecting, and a reference diagram. Two women opposed the term “girls” used as a colloquial term in the video by the lactation consultant. Other comments included that it was hard to read the accompanying text and watch at the same time, that there was a need for more eye contact, and that it was hard to hear in places (1.1% for all).

When specifically asked about the length of the video, 78.9% of women found it appropriate, while 17.9% found it too long and 1.1% found it too short. 

A total of 92 out of 95 women responded to the free text answer question about how they felt about the use of real women in the video, and all responded positively. Comments reflected women feeling more comforted about the process, even for those who had breastfed before, and finding a realistic approach helpful from a learning perspective (see Table 3).

A total of 93 out of 95 women responded to questions on video satisfaction which were determined on a continuous sliding scale of 1 (very unsatisfied), to 5 (very satisfied). All three aspects and overall satisfaction scored a median of 5.00, with a small interquartile range (IQR): depth of information covered (IQR 4.10–5.00, with 53.8% of respondents scoring 5), clarity of voices (IQR 4.10–5.00, 53.8% scoring 5), quality of images (IQR 4.25–5.00, 59.1% scoring 5), overall satisfaction (IQR 4.20–5.00, 53.8% scoring 5). Free text comments in this section indicated some minor dissatisfaction with the volume of voices, being told breastfeeding is best, a preference for non-scrolling text, a request for more information around how to administer the collected colostrum to a baby, the video being too long and a request for more specific information on hand placement.

When asked for additional suggestions for improvement of the video, three responses were received. These were the inclusion of a diagram of exactly where to place the hands with distance from the nipple and direction of pressure improvement, better music, and more focus on the syringe during the demonstration.

After viewing the video, approximately a fifth of women still had questions about ACE. Most questions related to safety, current health service recommendations for ACE and evidence around using ACE:

“Do all hospitals recommend doing this?”
“Can I start at 36 weeks?”
“Is it recommended for all uncomplicated pregnancies?”
“Have any studies been done on women who have previously experienced low supply to see if antenatal hand expressing helps?”
“Will this bring on early labour?”
“Will it help with establishing a good milk supply?”

This issue of safety and evidence can be better clarified as more research is done in the area.

Other questions focused on the use of stored colostrum, and have since been addressed in a revised version of the video:

“Should I give my baby the expressed colostrum even if he is feeding well?”
“How long can you store it frozen?”
“How do you defrost it?”

There were also questions specifically regarding expressing with gestational diabetes mellitus (GDM):

“What is the best time of day to express if you have gestational diabetes—does it matter if it’s done before or after a meal?”
“What are the benefits for GDM?”

In addition, one mother asked what to do if ACE was hurting, and another asked what to do if they try ACE and do not produce any colostrum.

After viewing the video, women were also asked whether they would be likely to recommend the video to pregnant family or friends, if hand expression of colostrum was suggested by their healthcare provider. Responses were gauged using a five-point Likert scale ranging from “definitely yes” to “definitely not”. Almost all women responded “definitely yes” (59.1%) or “probably yes” (37.6%), with 2.2% responding “might or might not” and 1.1% responding “probably not”. No women selected “definitely not”.

## 4. Discussion

The online video was effective in significantly increasing both knowledge and confidence around antenatal expressing of colostrum. The average knowledge score doubled in the post-video questionnaire, from 3.05 ± 1.70 to 6.32 ± 0.76, from a possible total score of 7. After viewing the video, the majority of women were able to correctly answer each knowledge question. The knowledge areas showing the largest improvements were when to start ACE (from 42.1% correct to 98.9% correct), and how often to express per day (from 26.3% correct to 94.7% correct). This observed increase in knowledge validates the most popular comments regarding what was liked about the video, which focused on the video being informative, detailed and interesting. If a learner finds the subject content interesting, this facilitates the learning process and makes them more likely to retain the information [10].

Reported confidence in using ACE also showed a substantial increase, from a mean score of 2.56 to 4.32 out of a possible 5. It is important to see an increase in confidence alongside an increase in knowledge, as knowledge alone is not necessarily enough to result in a change to practice. Self-confidence in applying skills, also known as a sense of competence, is recognised in the literature as being important when implementing health-related behaviour changes [11]. Importantly, self-confidence, along with expectations, can influence a mother’s judgement on her ability to breastfeed [12]. The use of real pregnant women in the video, as opposed to the use of breast models or actors, likely helped women to feel more confident—it was noted by the respondents as being the second most common liked aspect of the video. As in real life, the women in the video had differently shaped breasts and nipples, and this resonated with some respondents. Observational learning improves maternal breastfeeding confidence postpartum [12], and our results suggest that observational learning via video is also valuable during the prenatal period for improving confidence with hand expressing. Self-confidence encompasses both self-efficacy and self-esteem. Self-efficacy refers to an individual’s belief in his or her capacity to perform behaviours necessary to accomplish a specific task or achieve a certain outcome. Bandura’s theoretical sources of self-efficacy includes vicarious experience (alongside enactive mastery experience, verbal/social persuasion, and physiological and affective states), which refers to the learning of behaviour from observing others, such as watching videos of the behaviour [13,14]. This theory supports the concept that women who watch the ACE video and see women similar to themselves successfully expressing are more likely to succeed, due to an increased belief that they too possess the capabilities to master the skill of expressing in pregnancy.

The most common criticisms were that the video was too long and that it lacked some minor information, although these were an exception, with less than 20% of women expressing dislikes when prompted. When specifically asked about the length of the video, 17% reported that it was too long. The length of the video allowed for a complete demonstration and a relaxed atmosphere, which was noted as positive by some women, however it was possible to shorten it by speeding up some sections, particularly around the demonstration. Other aspects of video satisfaction indicated that it was well-received in terms of clarity of voices, quality of images and depth of information covered. 

We have since updated the video to cut out unnecessary and repetitive sections, reducing it to 15 min. We have also added additional information in the form of text notes at relevant times throughout the video.

Overall, 97% of women in this video evaluation stated that they would be likely to recommend it to pregnant family or friends, if hand expression of colostrum was suggested by their healthcare provider. Family and friend recommendations can have an important impact on public health strategies. Eighty-three percent of online respondents across 60 different countries said that they completely or somewhat trusted the recommendations of friends and family, according to Nielsen’s Global Trust in Advertising Report in 2015 [15]. The high level of recommendation demonstrated in our findings suggests that women valued the information they received from the video enough to share it with others. 

An online video provides unique advantages for instruction over a traditional face-to-face format. Being online allows pregnant women to view the video at a time and location convenient for them. This is particularly valuable for mothers who already have children, along with those located in remote areas. Another advantage of a video is the ability to pause and replay certain sections according to the viewer’s needs and understanding. Videos have become increasingly popular in education, and many universities now offer online instruction, incorporating videos and recorded lectures. Disadvantages also exist with video instruction, notably technical issues with glitches in internet bandwidth and connectivity [16]. In 2018, about 88% of the Australian population were active internet users, with an average of 5 h and 34 min spent on the internet daily [17]. A high-quality video with professional sound and lighting is key to better engage learners, as is audio/visual elements to the video that support the content above and beyond the use of text [18]. Results of our evaluation suggested that our ACE video is likely to be viewed as a valuable tool by pregnant women.

Risk of self-selection bias was an important potential limitation of this study, with women who were interested in ACE being more likely to participate. In addition, the women who participated in our study had a relatively narrow age range. These factors limited our ability to generalise our results to a wider population. 

Our findings have important implications for the use of ACE video instruction for future research, and potential public health initiatives leading on from this. Many mothers introduce formula due to a perception of poor milk supply [19], and cite a previously unsuccessful breastfeeding experience, poor breastmilk or difficulties breastfeeding as reasons for ceasing breastfeeding early [20]. A mother’s attitude towards breastfeeding has been shown to be a strong predictor of breastfeeding duration. This is shaped by personal factors including personal experience, exposure to positive role models and societal value [21]. Performing ACE and the storage of frozen colostrum in the weeks prior to birth has been demonstrated to build confidence with breastfeeding [22]. Instruction on ACE during pregnancy, alongside general instruction on breastfeeding routinely provided in antenatal classes, may help to build a positive attitude toward breastfeeding. Our video has been specifically designed for this purpose, and results from this study suggest that it is an acceptable and economical way to allow the efficacy and safety of ACE to be tested in future research. Future research may also investigate a potential role of this video in the education of healthcare practitioners working in maternal and infant health, who also have a need to increase knowledge, skills, and attitudes to assist breastfeeding families.

## Figures and Tables

**Table 1:** Characteristics of the pregnant women participating in the video evaluation (*n* = 95).

Characteristic/Response	Mean (SD) or Median (IQR)/*n* (%)
Age (years)	30.8 (4.7)
Gestation of current pregnancy (weeks)	31.0 (23.0–37.0)
Number of previous children:	
0	103 (60.2)
1	40 (23.4)
2	2 (12.3)
3+	7 (4.1)
Have previously breastfed an infant (yes)	56 (32.7%)

**Table 2:** Knowledge questions asked in pre- and post-questionnaires, with responses (*n* = 95). The related samples McNemar statistical test was used to compare the number of correct answers pre- and post-questionnaire for each question, and the paired *t*-test was used to compare the total knowledge scores.

Knowledge Questions and Responses * Indicates Correct Response to the Question	Pre *n* (%)	Post *n* (%)	*p*
If you wanted to do hand expressing in pregnancy, approximately when should you start? (with healthcare provider permission)			<0.001
After 28 weeks gestation	5 (5.3)	1 (1.1)	
After 32 weeks gestation	2 (2.1)	0	
After 35 weeks gestation	16 (16.8)	0	
After 37 weeks gestation *	40 (42.1)	94 (98.9)	
Not sure	32 (33.7)	0	
Click on the picture below that shows the correct finger placement for hand expressing (set of three photos displayed)			<0.001
Photo showing narrow finger placement	3 (3.2)	0	
Photo showing correct placement *	48 (50.5)	78 (82.1)	
Photo showing wide finger placement	16 (16.8)	17 (17.9)	
Not sure	28 (29.5)	0	
If you were doing hand expressing in pregnancy, how often should you express?			<0.001
Once per day	16 (16.8)	1 (1.1)	
Two or three times per day *	25 (26.3)	90 (94.7)	
Four times a day	2 (2.1)	2 (2.1)	
Not sure	52 (54.7)	2 (2.1)	
Can you reuse the same syringe for collecting colostrum over the day?			<0.001
Yes, can be left at room temperature	0	1 (1.1)	
Yes, as long as syringe is refrigerated *	30 (31.6)	91 (95.8)	
No, syringes are for a single expressing use only	30 (31.6)	3 (3.2)	
Not sure	35 (36.8)	0	
How much colostrum would you expect to collect in the first session of hand expressing?			<0.001
A lot (>10 mL)	0	0	
A moderate amount (between 1–10 mLs)	0	2 (2.1)	
A little (about 1 mL)	25 (26.3)	23 (24.23)	
Generally nothing at all	8 (8.4)	0	
Everybody will be different *	42 (44.2)	70 (73.7)	
Not sure	20 (21.1)	0	
If you can’t gather any colostrum during expressing, does this mean that you will have trouble breastfeeding?			<0.001
Yes	1 (1.1)	0	
Probably	3 (3.2)	0	
Probably not	13 (13.7)	9 (9.5)	
No *	53 (55.8)	85 (89.5)	
Not sure	25 (26.3)	1 (1.1)	
Should hand expressing be painful?			<0.001
Yes sometimes	7 (7.4)	1 (1.1)	
No *	52 (54.7)	92 (96.8)	
Not sure	36 (37.9)	2 (2.1)	
TOTAL KNOWLEDGE SCORE (mean ± SD correct/7)	3.05 ± 1.70	6.32 ± 0.76	<0.001

**Table 3:** Most common full text responses provided by pregnant women when giving their evaluation of the antenatal colostrum expressing video.

Response	Illustrative Quote
Aspects most liked about the video (*n* = 90/95 responded with full text response)	
The video was informative and interesting	*“Very informative, covering all aspects.”* (#44, 26 yo, 2 previous children, has breastfed previously) *“I found it very interesting and helpful. I will definitely be asking my midwife if I can do it when I turn 37 weeks.”* (#32, 36 yo, 1 previous child, has breastfed previously)
A practical demonstration using real women	*“Real women showing how to actually do it rather than cartoons or crochet breasts. Also that one woman had flat nipples like mine and was able to express which makes me hopeful.”* (#23, 30 yo, 1 previous child, no previous breastfeeding). *“It was good to see the spectrum of colostrum colour and volume. Good to see techniques. Good to see a primip and multip.”* (#52, 39 yo, no previous children). *“Close ups of colostrum and technique as well as how to collect on your own.”* (#60, 35 yo, no previous children).
Easy to understand	*“Easy to understand. And the first video I’ve watched where they’ve shown how to collect one handed using the syringe too.”* (#22, 32 yo, 1 previous child, has breastfed previously) *“Clear and explained well. Went over important bits twice”* (#21, 28 yo, 1 previous child, no previous breastfeeding)
Feelings about the use of real pregnant women in the video (*n* = 92/95 responded with full text response)	
More comforted about the process of antenatal expressing	*“I felt like I could relate to them more.”* (#32, 36 yo, 1 previous child, has breastfed previously) *“It was much more reassuring seeing real pregnant mums”* (#13, 33 yo, 2 previous children, has breastfed before) *“Comforting to know it was real people learning the skill”* (#73, 34 yo, no previous children)
A realistic approach	*“Excellent and helpful because both were different i.e.) different sized breasts, one had breastfed before the other was a first time mum”* (#1, 29 yo, no previous children) *“It was good to see colostrum actually being expressed rather than someone imitating the process be it through use of an actor or prop.”* (#76, 35 yo, no previous children).

# refers to subject number. Yo = years old.
